# Swarm Intelligence to Face IoT Challenges

**DOI:** 10.1155/2023/4254194

**Published:** 2023-05-29

**Authors:** Laith Abualigah, Deborah Falcone, Agostino Forestiero

**Affiliations:** ^1^Computer Science Department, Prince Hussein Bin Abdullah Faculty for Information Technology, Al Al-Bayt University, Mafraq 25113, Jordan; ^2^Institute for High Performance Computing and Networking, National Research Council, Rende, CS, Italy

## Abstract

The Internet of Things (IoT) paradigm denotes billions of physical entities connected to Internet that allow the collecting and sharing of big amounts of data. Everything may become a component of the IoT thanks to advancements in hardware, software, and wireless network availability. Devices get an advanced level of digital intelligence that enables them to transmit real-time data without applying for human support. However, IoT also comes with its own set of unique challenges. Heavy network traffic is generated in the IoT environment for transmitting data. Reducing network traffic by determining the shortest route from the source to the aim decreases overall system response time and energy consumption costs. This translates into the need to define efficient routing algorithms. Many IoT devices are powered by batteries with limited lifetime, so in order to ensure remote, continuous, distributed, and decentralized control and self-organization of these devices, power-aware techniques are highly desirable. Another requirement is to manage huge amounts of dynamically changing data. This paper reviews a set of swarm intelligence (SI) algorithms applied to the main challenges introduced by the IoT. SI algorithms try to determine the best path for insects by modeling the hunting behavior of the agent community. These algorithms are suitable for IoT needs because of their flexibility, resilience, dissemination degree, and extension.

## 1. Introduction

In 1999, professor K. Ashton of the Massachusetts Institute of Technology (MIT) introduced the notion of the Internet of Things (IoT). IoT is the combination of physical and digital objects embedded with sensors, actuators, network connectivity, and processing ability to produce new goods/services and allow creative business strategies. It is now possible to digitize features and essential capabilities of industrial-era goods because of advancements in microprocessor technology, broadband connection, dependable memory, and power management [1]. As a result, a variety of opportunities are emerging for businesses to provide additional value in the IoT. IoT solutions are quickly being used in almost every aspect of daily life, and their range of applications is as broad as it is varied. The most common application areas are in the fields of smart industry, smart buildings or homes, smart energy, smart transportation, and smart health [2].

An IoT technology infrastructure typically consists of three fundamental layers: (i) the device layer, (ii) the connection layer, and (iii) the cloud layer. A variety of smart devices, such as sensors, actuators, cellphones, and mobile and wearable gadgets, constitute the first layer. At the connection layer, communication protocols make it easier for individual entities and the cloud to communicate. Device-to-(device, cloud, gateway) and back-end data sharing are several smart object communication paradigms. Because of its simplicity of use, flexibility, and scalability, cloud computing is recognized as the cornerstone. To interact with administer and manage connected items at the IoT cloud layer, device communication and specific implementation are employed, while an application platform enables the creation and usage of smart applications. Moreover, data produced by connected objects are utilized to lead with an analytical and database management system. Massive volumes of data may be organized, analyzed, and evaluated by engineers and data scientists thanks to artificial intelligence (AI) technologies. The area of computer science primarily responsible for and capable of making big breakthroughs is artificial intelligence (AI), which makes it possible to extract useable information from the vast, intricate, and real-time data produced by IoT devices.

Despite the achievements revealed in using IoT, there are a number of concerns about the risks in the growth of this technology and its products. The variables that need to be looked at include device heterogeneity or interoperability, scalability, widespread usage of wireless technology, optimal energy consumption, resource constraints, data management, privacy preservation, bandwidth, data rate, and latency. Some of the technologies that can be employed in this scenario are communication technologies like Wi-Fi, radio-frequency identification (RFID), and Bluetooth. IoT is primarily enabled by wireless sensor networks, which are key technologies (WSN). Interconnected sensor nodes, WSNs, use wireless communication to exchange information about their surroundings. Nodes are often dispersed randomly and without any centralized control. One of the major problems affecting this type of network's performance and requiring more attention is how routing is implemented. Routing, in general, is the process of finding, choosing, and maintaining pathways for transmitting data packets from a source to a destination host. Every routing algorithm aims to maximize network performance while minimizing costs by directing traffic from sources to destinations. The energy consumption of smart devices is another crucial issue that must be addressed. Even if IoT devices are equipped with increasing computing power, remote and continuous monitoring capabilities, and big data analytical skills, most of them are battery-power operated. Therefore, defining power-aware strategies to address the problem of energy capacity shortage must be one of the main design principles in IoT systems. Moreover, smart IoT devices generate high volume, velocity, variety, and veracity data. Data processing methods may experience performance degradation and a corresponding increase in processing time with such a vast volume of data. As a result, data processing, storage, and presentation also contribute to IoT value.

To mitigate and often solve many of these problems, one approach is the application of swarm intelligence, or SI, in the IoT. The main goal of swarm intelligence is to artificially recreate the idea of naturally intelligent, decentralized creatures, where the group's collective intelligence is greater than the sum of its members' individual intellect. Based on this idea, algorithms for complicated systems were discovered, including ant colony optimization, bacteria foraging, bird flocking, and fish schooling [3]. Due to their potential parallelism and decentralized properties, SI algorithms have improved self-adaptability, robustness, and searchability properties, enabling them to address challenging nonlinear problems. It may be used to solve NP problems as well as a variety of optimization problems, such as dynamic optimization techniques and multi-objective optimization issues [4, 5]. SI has excellent application potential in IoT-related applications, owing to the ongoing growth of smart environments [6].

The goal is to simplify a complicated or more significant job by adopting an SI-based algorithm for achieving global optimization by modeling the IoT network as a collection of essential devices carrying out certain simple functions. The objects cooperate utilizing decentralized control and self-organization in accordance with the SI tenets. A system becomes more effective, efficient, and scalable when a distributed type of control is used. SI is applicable to cloud computing scenarios, which makes it easier to solve multi-objective optimization issues. IoT data processing might become considerably quicker and more effective with its use. Once more, SI can address critical issues including cluster head selection, node localization, and routing protocol planning. IoT ecosystems would benefit from SI-based techniques in IoT-based systems, which would also enhance the user experience for IoT apps and services.

The contribution of the paper is to summarize SI-based algorithms applied to the IoT for solving the main challenges of this technology. We have classified a rich set of methods on the basis of the main SI algorithm employed, i.e., ant colony optimization (ACO), particle swarm optimization (PSO), and bee colony optimization (ABC), with respect to three main IoT concerns: (i) routing protocols and CH selection, (ii) power management, and (iii) data management.

This paper is structured as follows.  Section 2 introduces the main SI algorithms.  Section 3 presents the application of swarm intelligence algorithms to address IoT challenges.  Section 4 discusses future directions and challenges before concluding the paper.

## 2. Swarm Intelligence Algorithms

The study of large-scale, distributed systems that coordinate through decentralized control and self-organization, such as those seen in nature and in artificial systems, is known as swarm intelligence. Those who are looking for solutions get knowledge from other people's experiences. Collectively adjusting to the local and/or global environment maximizes a function or objective. The idea of a swarm implies diversity, stochasticity, unpredictability, and disorder, but the idea of intelligence implies that the approach to problem solving is somehow effective. The people that make up a swarm can be real or imagined, living, mechanical, computational, or mathematical, including (i) insects, birds, or humans and (ii) array elements, robots, or isolated workstations. There has to be an interaction between the persons for their connection to have a variety of features [7].

In [8] are defined the following SI basic principles:

- Proximity principle: the general people should be capable of doing basic space and time calculations.
- Quality principle: the general people should react to environmental goodness variables.
- Diverse response principle: the general people should not conduct their affairs within too limited routes.
- Stability principle: the general populace should not alter its behavioral pattern in response to environmental changes.
- Adaptability principle: individuals in the population must change their behavior when doing so is worthwhile from a computational standpoint.

There are many SI-inspired optimization algorithms; the most popular are the ant colony optimization (ACO), the classical particle swarm optimization (PSO), and the artificial bee colony (ABC). As stated in [9], several SI-based techniques have been created based on various intelligent behaviors of cooperating multitudes found in nature, including those of ants, pigeons, fireflies, bees, birds, and bats. These algorithms include firefly optimization algorithm (FFA), honey bee mating optimization (HBMO), mosquito host-seeking algorithm (MHSA), glow-worm swarm optimization algorithm (GSO), social spider optimization (SSO), slime mould optimization algorithm (SMOA), pigeon-inspired optimization (PIO), bat algorithm (BA), cuckoo search algorithm (CSA), shuffled frog leaping algorithm (SFLA), artificial fish swarm algorithm (AFSA), frog calling algorithm (FCA), grey wolf optimizer (GWO), roach infestation optimization (RIO), fish school search (FSS), monkey algorithm (MA), lion pride optimizer (LPO), dolphin echolocation algorithm (DEA), lion optimization algorithm (LOA), and cat swarm optimization (CSO). SI algorithms all have the traits of being population-based, iterative, and inspired by animals. Their examination of the workstation, though, varies. Here, based on previously available scientific material, we describe pertinent applications of the algorithms. This part briefly overviews the most popular biologically inspired swarm intelligence algorithms.

### 2.1. Particle Swarm Optimization

Including insects, cattle, birds, and fish, the particle swarm optimization (PSO) technique aims to simulate colonial behavior in collective animals. These swarms adopt a cooperative approach to food gathering, and each swarm member continuously modifies the search pattern in response to its own and other members' growing experiences.

The early simple model of the algorithm, namely, the Boid model [10], is designed to simulate the behavior of birds. Based on this model, each bird has an initial velocity and position. According to the “next proximity velocity match rule,” each person moves at the same speed as its closest neighbor. All of the points' velocities will eventually be equal if the iteration is continued in the same way. To replicate actual-world circumstances, each speed will be doubled by a random variable. Afterward, Russell and Kennedy [11] defined the particle swarm optimization (PSO) algorithm. They reduced each individual to a particle with no mass or volume, only velocity and location. Each entity in the swarm follows three major rules: (a) avoid collisions with nearby entities; (b) match its own velocity with the vicinity; and (c) fly to the flock's epicenter, and the swarm overall flies to the target. The PSO algorithm is a swarm-based method of searching in which each component is referred to as a particle and is represented as a reasonable solution of the optimization problem in a D-dimensional examination space. It is capable of memorizing both the multitude's ideal position and its own, in addition to the velocity. The particle data are combined in each iteration to change each dimension's velocity, which are then used to determine the particle's new position. Particles in the multi-dimensional search space continually alter their states until they attain a balance or ideal state or exceed the calculating boundaries. The goal functions to introduce the unique relationship between several aspects of the issue space. A large body of empirical evidence has proven this method to be an efficient optimization technique.

### 2.2. Ant Colony Optimization

The ant colony optimization (ACO) technique is inspired by how ant colonies optimize their paths to food sources. One of the greatest illustrations of a natural swarm is an ant colony. Each ant sprays pheromones along the trail from their nest to the food source. After a while, the whole ant colony will be capable of taking the shortest route to the food because the ants in the colony will select the way with the highest pheromone intensity, and the ants moving along the path will create pheromones along the way. Solitary ants lack the intelligence to determine the quickest route to their meal. Nonetheless, they can readily complete a variety of difficult activities while living in colonies. ACO algorithms are used to solve optimization, traveling salesman, scheduling, and vehicle routing difficulties, among other challenges. Strong global optimum capability and flexibility in deployment are two benefits of ACO. The pheromone level is updated as the primary iteration in the ACO. It can be used in conjunction with other algorithms.

### 2.3. Bee Colony Optimization

The bee colony optimization (ABC) algorithm mimics a swarm of bees' honey-gathering activity. Another excellent example of a biological and dynamic swarm is a hive of bees. By distributing their labor among other bees, they demonstrate their intelligence. Bees exhibit various behaviors per their individual work divisions and understand the need for bee swarm communication and exchange of information to arrive at the best outcome. They carry out activities that include hunting, gathering, storing, distributing honey, gathering pollen, retrieving, and responding to changes. The algorithm used to create the simulated bee colony separates the bee multitude into productive and jobless bees. When a food source is discovered among the latter, jobless bees scout it out. Worker bees randomly wander from one blossom to the next in search of a food supply. The unemployed bees locate the closest route to the food source after it has been identified, and when they do, they alert the other agents by doing a waggle dance. Employee bees gather nectar at the source and then take it back to their comb to store it. Each food source symbolizes a potential answer to the issue, and the quantity of nectar emanating from the source suggests the potency of the answer. The function of the bees may be switched around in the artificial colony of bees approach, which makes it different from prior SI algorithms. For instance, it should be stopped if the source data do not change after multiple rounds. ABC's benefits include its excellent global search capability and rapid convergence speed. Its downsides include a lack of racial variety. It is possible for the solution to enter the local optimum when it is close to the global optimum, resulting in a situation of stagnation [6].

## 3. IoT Challenges Faced by SI

The Internet of Things has transformed the traditional way of life into a modern one. Smart cities [12–14], smart homes [15, 16], smart health [17–19], smart industries [20], smart transportation [21, 22], agriculture [23, 24], energy saving [25], and pollution control [26] are such transformations due to IoT. Several important research projects and studies have been carried out in an effort to advance technology through IoT. To fully realize the promise of IoT, several obstacles and problems must be resolved. Standard communication protocols must be adopted for effective device integration, yet IoT devices do not adhere to the same standards and protocols. Because of this, the nonuniformity of their qualities makes data aggregation more challenging, slowing down the entire process and reducing IoT scalability. The volume of recorded data is growing as a result of linked device proliferation, high-resolution instrumentation, and big populations. Computer scientists face enormous hurdles due to the surge in data generation, including data storage, access, visualization, analysis, and modeling issues. Although we can typically reduce and resolve issues like scalability, resource constraints, and data management using cloud computing, parallel computing, and artificial intelligence, more work still needs to be done.

Cloud computing and IoT are separate yet robust systems integrated to become essential to the Internet's future. The idea behind this integration is that IoT devices are networked sensors designed to collect data and transmit it to the cloud for computing. An important requirement is to provide reliability and low-latency analysis offloading, enabling forthcoming IoT services with stringent requirements [27]. Because of the considerable amount of confidential data stored in the cloud, data protection and privacy are major unresolved concerns [28].

The majority of smart devices lack information standards and protocols, and the laws governing data ownership are unclear. Due to all of these variables, the data are quite vulnerable to hackers who can compromise personal information by breaking into the system. In addition to other things, the power management of smart devices is a significant problem because of the limited capabilities of the devices and the high application needs. Most IoT devices are battery-powered, which provides essential benefits in terms of mobility, freedom of installation, flexibility, and ease of use; however, the harder an IoT device has to work, the more power it will draw.

Swarm intelligence is an excellent ally to deal with some of the problems that IoT technology's maturation brings. For instance, a model called DF-IDS is proposed in [29] and is modeled on spider monkeys' foraging behavior. Via two primary steps, it seeks to find IoT traffic intrusions. In the first step, it competitively chooses the most suitable components from the characteristic matrix utilizing SpiderMonkey (SM), principal information gain (IG), component analysis (PCA), and correlation attribute assessment (CAE). These attributes are employed in the second step to train a deep-NN for intrusion detection. In [30], the authors applied a distributed hashing model to perform the goodness of large amounts of data collected by devices, bearing in mind their processing and power capabilities. This section offers an overview of SI algorithms applied to three main IoT challenges: (i) routing protocol planning and cluster head selection, (ii) power management, and (iii) data management.

### 3.1. Routing Protocols and CH Selection Methods

A considerable percentage of SI-based routing protocols have emerged in wireless sensor networks (WSNs). Increasing performance, preserving energy, and ensuring secure communication are the main goals of WSN design—all essential components of a reliable IoT system. The WSN-IoT provides opportunities and difficulties. Many SI-based techniques have been created in accordance with the types of WSN-related challenges. Due to the extremely huge scale of WSN, where nodes are frequently installed at random, it is necessary to take into account the effective design of communication protocols in such networks. Protocols must be resilient to errors and losses as well as self-organizing. Moreover, a well-planned routing protocol allows saving energy, which is a key requirement in WSN.

Routing protocols in WSNs may be divided into flat protocols and vertical routing methods based on communication logic [31]. Networks are divided into clusters of varying sizes in hierarchical routing protocols, and cluster head (CH) identification is a critical topic. Each cluster is made up of a CH and many cluster components. The representative of the cluster oversees or controls all of the cluster's nodes, coordinates activity among member nodes, and is in charge of information collecting in the cluster, data fusion processing, and cluster forwarding. As a result, CH energy consumption is often higher. The WSN relies on optimum routing methods to preserve communication energy and hence extend the network lifespan.

A broad overview of algorithms that concentrate on this problem is presented in [6, 9]. Based on these references, we have classified routing protocols and CH selection methods on the basis of the SI algorithm employed, as described in Table 1.

#### 3.1.1. PSO-Based Algorithm

The authors in [32] proposed a PSO and Tabu search-based WSN routing optimization method. With the aim of increasing the network lifetime, the Tabu-PSO method tries to maximize the number of clusters and nodes in each cluster. The CH in the cluster that uses the least energy is also picked. The findings show that increasing the amount of clusters and boosting node survivability can dramatically lower average end-to-end latency and mean packet loss rate.

Reference [33] presented the EMSP routing method, which incorporated artificial clustering approaches and mobile source technologies in the routing management. For selecting CH, the algorithm considers residual energy and node position (distance node - center of gravity).

The mobile sink selects the node with leftover energy that is more than the mean residual energy of all the cluster's networks as a new CH after examining all cluster heads. The group with the most energy remaining is where the node initiates collecting data from. The member node will not transmit messages to the CH until the sink has determined which CH it is. In this case, it is very likely that the member node will fail first before any data are sent. According to the data, both the average delivery delay and network life have improved.

Another PSO-based CH selection strategy and a cluster creation based on weight operation are proposed [34]. In addition to the commonly used distance from non-CH to CH, the cluster formation technique also takes into account the distance from the access point to CH, the CH's distance, and the CH's remaining energy. The technique takes into account WSN capacity balancing and fault tolerance and only operates on isomorphic networks. Following these concepts, a method based on fuzzy clustering and PSO was proposed in [35]. The overall number of disconnected sensors for all clusters is what the PSO algorithm uses as its basis for trying to find CH. In order to minimize network interruption, the best CH is then used to update the fuzzy C-means (FCM) algorithm. In order to determine the optimal sensor topology, the hybrid FCM-PSO approach is applied. The quantity of sensors that cannot be linked to the CH and the proportion of CHs that cannot contact the station in 3D-WSN can both be decreased, according to simulation results, using this technique.

In [36], a PSO-based clustering technique (EC-PSO) in a heterogeneous environment is described. The network's energy center is located using the PSO, and the nodes that are closest to it are selected as CHs. The recommended technique states that CH should be located in the energy center since CH and the nodes around it use a lot of energy. Energy holes are prevented from forming through clustering. A low-energy protection mechanism was created, and a mobile data collector was set up to collect data in order to prevent the weak node from turning into a relay node. The technique performs well in terms of energy usage.

An optimization method based on the improved PSO algorithm was applied in [37] to extend the target nodes throughout the network life cycle. The protocol accounts for both transmission distance and energy efficiency, and relay nodes are employed to reduce the cluster heads' excessive power consumption. Using the suggested protocol, the network's lifespan is increased through more evenly distributed sensors and a balanced clustering scheme.

In [38], in order to build, recover from, and choose k-disjoint paths that allow failure while preserving the quality of service features, authors provided a bio-inspired PSO-based routing method. By exchanging messages from all locations in the network, the approach enables identifying the optimum routes while using multi-path routing. Compared to the standard PSO, the overall energy consumption and latency are decreased. Because the method takes a while to stabilize the feasible solution in the start of the iteration, there is a little disadvantage.

When a path fails in the Internet of Things, the authors in [39] propose an enhanced, effective, and adaptive fault-tolerance routing algorithm (IEIFTA) to offer quick routing recovery and reconstruct the network architecture. With the use of a multi-swarm evolution equation, the IEIFTA, a PSO-based method, determines the direction in which the particle will mutate. The immune mechanism boosts the efficiency of global search and the algorithm's rate of convergence.

#### 3.1.2. ACO-Based Algorithm

In order to address the routing and CH selection issues, several ACO-based methods are developed. A routing method that incorporates ACO, the mobile sink, and the clustering algorithm was proposed by authors in [40]. The algorithm uses the modified ACO to choose the most effective sink movement path, communicate with all CHs, and collect information via a single-hop closer contact. The heuristic factor is maximized to achieve this (visibility). This method, which only rotates CH whenever a CH remaining energy does not exceed the energy threshold, can increase the network life compared to earlier ACO-based standard algorithms.

A cross-layer protocol (FAMACROW) was proposed in [41]. Fuzzy logic is employed in this method to choose CH, and ACO technology is utilized to perform multi-hop routing from a cluster to the master station. The approach employs connection quality indicator, neighborhood proximity, and residual energy as input parameters. Using connection quality metrics to choose CH and transmit the likelihood of routes between clusters increases the dependability of protocols. In terms of latency, throughput, and network life, FAMACROW outperforms the other uneven clustering routing.

In [43], the authors proposed an ACO-based routing algorithm in order to determine the WSNs' ideal route for data transmission. The algorithm uses an improved heuristic function and considers location information and search direction. In particular, it takes into account both the distance between one node and its sink as well as the distance between nodes. When the sink and the objective region are far apart, the method performs noticeably better than previous algorithms.

Another routing protocol, named SRPMA and based on ACO, is proposed in [44]. This method seeks to improve multi-path routing outcomes. The primary idea is to incorporate the Pareto multi-objective approach with the ACO. As optimization targets, the lifetime of devices and the reliability of a routing pathway are considered. The node trust management model is developed by evaluating the node's trust level using an upgraded D-S evidence model with preprocessing. In WSN routing, the SRPMA can achieve performance requirements against the black hole attack when compared to similar ACO-based security routing protocols.

In [45], ACO was combined with a label propagation (LP) algorithm. CB-RACO is the proposed routing protocol's name. High data transmission speeds are desired for large-scale WSNs. Even though this protocol divides clusters and is not CH-based, each ant can only reside in its own cluster (cluster activity). The benefit of this method is that it can manage and operate the network independently when it is overloaded and at a very cheap memory cost.

The authors of [46] presented an ACO-based routing mechanism to improve packet delivery speed and prevent overlapped junctions by utilizing multi-agent technology. According to the network type, the proposed method separates the IoT ecosystem into different sections. The ACO algorithm that is most suited to each network is then chosen. A dual entity is created to generate an optimum algorithm from various ACO methods.

Similarly, the IoT environment in [47] is separated into segmented zones based on network types. The most appropriate ant colony algorithm is then applied to the relevant network inside each location. Each network is assigned an ACO in charge of controlling the routing process. The authors suggested a technique for regulating the employment of ACO algorithms and figuring out a solution for covered regions. The routing algorithm's goal is to optimize the choice of the most suitable path for data transmission inside the IoT system.

In [48], ACO was integrated with RPL-IPv6 routing protocol. RPL offers a 6LoWPAN abstraction layer routing solution that uses the least amount of energy possible for the sensors. In order to maximize path power usage from over-expected transmission cost metric-based cost function, which represents the sum of energy consumption by the transmitter during communication and the listening base station during listening, the proposed approach offers an approach to switch from the failing connection to another effective link.

A hybrid multi-path routing algorithm was proposed in [42]. The approach is based on the joint use of ACO and ABC and is called exponential ant colony optimization (EACO). The fractional ABC (FABC) method is used in the first phase to find CHs with a fitness function that takes distance, power, and delay into account. The ACO method is altered with an exponential smoothing model supporting multi-path route finding in the second phase. In order to move data from any source address to the gateway destination with the least amount of energy, location, and intra/inter-cluster delay, EACO determines the best pathways among CHs. These goals are well-stated as new fitness metrics to choose the best course of action.

In [49], the optimal cluster head was selected from a group of nodes using the butterfly optimization algorithm (BOA). The distance to neighbors, the distance to the access point, the node degree, and the node centrality all have an impact on the cluster selection. ACO is exploited to determine the path between the cluster and the base node. It chooses the best path based on the distance, remaining energy, and node degree. The energy use, data packets obtained by the BS, living nodes, dead nodes, and other performance metrics of this suggested approach are examined. The study's goals are to reduce total energy usage and increase network longevity.

#### 3.1.3. ABC-Based Algorithm

A centralized clustering-based routing protocol exploiting the ABC to adjust fuzzy rules was proposed in [50]. The outcomes demonstrate that the LEACH-SF algorithm is capable of minimizing distances inside clusters while maximizing network lifespan and base station packet reception rates. Moreover, it possesses the ability to be enhanced for multi-hop routing and mobile sensor nodes. On a heterogeneous network, the simulation is built.

In [51], the authors suggested a method for choosing the most significant cluster heads. They empower the ABC algorithm with the gravitational search algorithm (GSA). Until it achieves the halting condition, GSA updates the agents' positions and speeds. In this situation, the ABC algorithm's updated employed bee phase is updated using the GSA algorithm. During the selection of cluster heads, the IoT devices' distance, energy, latency, load, and temperature are taken into account.

This section collects numerous SI-based algorithms to address the problems of routing and CH selection in IoT environments. These algorithms explicitly or implicitly aim at decreasing network energy consumption, which is one of the main problems for such systems. The next section focuses on algorithms that offer power management techniques in IoT.

### 3.2. Power Management Methods

The energy management of mobile devices, sensors, wearable technology, and other IoT items is crucial in IoT contexts. As IoT devices run on batteries and energy conservation has emerged as one of the industry's top issues, this section examines several strategies centered on that issue. We discussed different energy-efficient and SI-based techniques proposed in state of the art. Most of the following references focused on energy-constrained IoT-based WSNs. An active study area in IoT-based systems is extending the network's lifetime while attaining optimal coverage WSNs. It is much more challenging than any other kind of network.

In Table 2, power management methods are classified on the basis of the SI algorithm employed.

#### 3.2.1. PSO-Based Algorithms

Energy-efficient communication is the goal of IoT. Bluetooth Low Energy is an intriguing option for wireless communications. The Bluetooth 5 standards were recently released with the intention of providing notable improvements over the protocol's prior iterations. In [52], to deal with choosing the Bluetooth 5 connection mode that provides for the highest energy efficiency, the authors proposed a fuzzy-based method. The idea is to adjust a fuzzy logic controller's transmission power output in order to dynamically control the communication mode utilized by mobile devices (FLC). To determine the optimal performance conditions values for the suggested FLC, a PSO method is described. In particular, PSO enhances triangular membership characteristics by modifying their range to obtain the best results for mobile device battery capacity. Triangular membership functions are considered the foundation of the proposed FLC because they offer a reasonable balance between computational cost and effectiveness.

Energy harvesting techniques are significant IoT power management research areas. The energy-collecting device may directly tap into the immediate environment, including pressure, vibration, wind, temperature gradients, and the sun's energy source. In [53], the authors have demonstrated a brand-new, enhanced power management unit (PMU) that is suitable for IoT applications and may be included in an energy collection system. Solar cells, turbines, thermal, and vibration may all be included in the system. Through the use of a buck-boost DC-DC converter, the optimized PMU is created. The PMU's characteristics, with the converter and control circuits, are then optimized using the PSO method to increase overall power efficiency and reach maximum power point. The primary goal of using PSO is to choose appropriate inductor and MOSFET on-time values while computing and simulating power loss equations to reduce the energy consumption of the inductor, photodiode, MOSFET, and management system.

In [54], a PSO-enhanced IOT-based power point tracking (MPPT) method was suggested for solar photovoltaic (PV) systems. The most promising renewable energy source is the photovoltaic system, and tracking of maximum power points (MPPT) is crucial to the smooth operation of any PV-based powered system. Smart meters, sensors, and actuators are all incorporated into IoT-based technologies to enable MPPT operation of the photovoltaic electricity system. They enable distributed connection and automation for management and oversight of the MPPT system. The antenna receives processed sensor data. Moreover, IoT helps dynamically identify the MPP region of solar modules. In this study, a modified DC-DC ZETA converter is an interface between the DC load and the solar PV. The PSO-IoT method uses Arduino and Bluetooth to continually modify the converter's duty cycle in order to gather the most power possible. The suggested PSO-IOT MPPT approach has a high PV tracking efficiency and, in comparison to other optimization techniques mentioned in the literature, converges quickly with little variations around Global MPP (GMPP).

Hurtado et al. [55] proposed an agent-based strategy to improve how the SG-BEMS framework interacts with one another. It is necessary to go from a “vertical” to a “horizontal” form for future power systems. As buildings account for a large portion of total energy consumption, it is crucial to manage the futuristic power grid and built environment in a way that promotes energy efficiency and environmental responsibility. A sophisticated building energy control system (BEMS) is needed to handle the extremely complicated interactions between two environments throughout this evolution to a smart grid. A PSO optimizer is suggested in order to increase multi-agent systems' (MAS) capacity for taking use of the building's adaptability for the smart grid. PSO is employed to increase comfort and energy effectiveness. According to a numerical result from an integrated simulation, the building's operation may be dynamically changed to help the local power grid's voltage management without impacting the building's principal function, namely, comfort provision.

#### 3.2.2. ACO-Based Algorithms

With the help of a brand-new ACO algorithm called three-pheromone ACO, Lee et al. [56] provided a new method to address the efficient-energy coverage issue in WSN (TPACO). In contrast to existing ACO algorithms, which only use one form of pheromone, the proposed ACO algorithm uses three different kinds of pheromones to identify the solution successfully. In contrast to the other two pheromones, which are used to form a collection of sensors that contains as many sensors as the ant has selected the number of active sensors to be employed, the local pheromone assists the ant in organizing its coverage set so that it employs fewer sensors. The TPACO algorithm also has the advantage of not requiring the two separate user variables of the ACO algorithm. The authors also presented two methods that help address the efficient-energy coverage challenge in a more practical way. The probability-based sensor detection model is used as the first method. The second technique involves using many types of sensors, or heterogeneous sensors, in continuous space rather than a discrete region based on a grid. Results from the simulation show that the recommended technique is effective in terms of network longevity.

With the same aim, in [57], in order to discover the best route for recruiting sensor nodes for signal transmission, a customized version of the ACO algorithm is used. The intra-node spatial range and the speed of battery fall out/recovery with regard to signal transmission are combined in the suggested technique.

#### 3.2.3. ABC-Based Algorithms

In [58], for finding the ideal number of disjoint subsets, the HABCA-EST method, a hybrid ABC algorithm including an efficient schedule transformation, was presented. The objective is to extend a wireless smart device network's life for applications that require target coverage. Nondeterministic polynomial completeness characterizes this issue. The distinguishing characteristic of HABCA-EST is the explosive development of the fitness function as a result of the full exploitation of surplus information among the planned devices. In HABCA-EST, the swarm and EST processes cooperate to find an ideal solution in less time efficiently.

In [59], an energy-efficient clustering mechanism was developed. It consists of two primary stages. The ABC method is used in the first step to choose the nearly ideal cluster heads. Performance requirements for a device include its remaining energy, the quantity of nearby devices, the Euclidean distance (ED) between it and the source, and the ED from it to each of its neighbors. The principal objective of the second phase is to cluster devices into a small number of groups based on the amount of data that each group produces, as well as the ED between each group's leader and members. It has been proven that the authors' approach is effective in terms of energy consumption, durability, and transmission delay.

The same objective drove the work done in [60, 61]. Distributed mobile sink-based method for collecting data, which combines energy-balanced clustering with ABC-based data gathering, was proposed by the authors in [60]. The cluster head is determined by the node's remaining energy. They looked at mobile sink balancing from three angles: maximizing data gathering, minimizing mobile route length, and improving network dependability. Simulation findings demonstrate that, in contrast to existing algorithms like random walk and ACO, the recommended approach may effectively reduce data transfer, save energy, improve the accuracy and dependability of network data collection, and enhance network lifetime.

To strengthen the suggested metaheuristic's worldwide convergence and to improve its exploitation capabilities, in [61], an enhanced ABC (iABC) evolutionary algorithm with a better search equation was designed. Student's T-distribution is used to create significant sampling techniques since it only needs one regularization term to be computed and stored. To create the best cluster heads and boost WSN energy efficiency, the iABC optimization algorithm is also employed to develop a power bee clustering protocol. This protocol inherits the efficacy of the recommended metaheuristic. According to the findings of the simulation, the suggested strategy performs well in terms of availability, packet delivery, use of energy, and network durability.

### 3.3. Data Management Methods

The volume of recorded data is growing as a consequence of the expansion of IoT-linked infrastructures, high-resolution equipment, and big populations. Computer scientists face enormous hurdles as a result of the surge in data generation, including issues with data storage, access, presentation, analysis, and modeling.

Data management and mining technologies are fundamental in various IoT applications; as a result, it is possible to develop more robust models, and swarm technologies can assist with this. In Table 3, we have classified data management methods on the basis of the SI algorithm employed.

#### 3.3.1. PSO-Based Algorithms

The use of data mining and intelligent systems for IoT was discussed in [15]. Each sensor is connected to a straightforward agent in the authors' sophisticated data management platform founded on swarm optimization. ACO and PSO have an impact on how each agent senses. By sharing information and experience among themselves, agents act like a swarm. To further explain the fundamental ideas of DMFSO, the authors utilized a straightforward example of a smart house to demonstrate information sharing and decision making.

A PSO-based backpropagation (BP) artificial neural technique to large data mining is suggested by Zhou et al. in [62] for economic risk management in financial institutions with a smart implementation. Using the collection of on/off-balance sheet items, it constructs a nonlinear parallel optimization approach using Apache Spark and Hadoop HDFS methods. The experiment's findings show that this concurrent risk management model performs well at screening default behaviors and has a quick convergence rate and predictive solid capacity. The distributed implementation on significant data clusters in the meantime has dramatically reduced the processing time for model training and testing.

To effectively increase the measurement precision of physiological multi-sensor data fusion in IOT, an enhanced PSO approach (IPSO) is presented in [63]. IPSO incorporates shrinkage factor adjustment and inertia weight factor design. The multi-physiological data processing and real-time medical care of things analyses are built on the Android platform.

#### 3.3.2. ACO-Based Algorithms

The authors in [65] proposed using ACO for data optimization to shorten the time required for processing all of the data. For quick data mining on larger datasets, ACO provides large-scale optimization. It can process high-dimensional data to ensure that the performance of the algorithm is not adversely affected by massive datasets, and it can handle dynamic data, enabling practically real-time data processing. It supports multi-objective optimization in ACO, which enables the management of data from various sources. This is a reliable and effective method for IoT data optimization.

In [64], an ACO-based clustering method (ACOCA) is suggested for massive data preprocessing. The hybrid algorithm can facilitate faster search by streamlining the procedure. The combined use of ACO with the clustering method further speeds up preprocessing and improves the precision and effectiveness of analysis.

In order to increase the exploitation of agricultural big data and address the security concerns associated with multi-source and heterogeneous agricultural big data, the authors in [24] suggested an enhanced agricultural big data ACO algorithm (BigDataACO). The approach tackles the issue of multi-source data fusion and offers excellent resilience and parallelism potential. Findings demonstrate that the improved method described in this research significantly reduces the uncertainty of data fusion when compared to D-S evidence theory, K-means, and Bayesian algorithm.

In [13], in order to automate data processing and provide useful information to be utilized in the autonomous management of traffic circulation in major metropolises, the authors addressed the issue of collecting data from many sensors. They presented a brand-new platform created for the intelligent administration of automated environments by heavyweight agents and the integration of diverse sensors utilizing lightweight agents. These later ones have information fusion expertise. One of the ACO models, H-ABC (hierarchical ant-based control), is used by specialized agents to carry out the required data transformations.

#### 3.3.3. ABC-Based Algorithms

Due to its advantageous characteristics, such as having few controllable parameters, being very flexible, and having a powerful global search capability, ABC has been exploited to solve several optimization issues [66]. Large datasets are processed and features are chosen using it as well. A Hadoop-based ABC (H-ABC) strategy for feature extraction in medical big data IoT was presented in [19]. ABC is used with traditional MapReduce to enhance processing efficiency. A parallel technique that supports MapReduce effectively handles a huge number of datasets. Using 10 distinct datasets, the suggested method is compared to swarm techniques and assessed in terms of effectiveness, accuracy, and throughput. The findings demonstrate how the suggested approach is more scalable and effective in choosing characteristics.

## 4. Challenges and Future Directions

IoT is a paradigm that demands energy savings and real-time management or a scenario with a quick reaction and high transmission rate. IoT settings are intricate, sizable, and distributed systems that face difficulties with heterogeneity, security, and dependability. They must also consider a number of challenges related to efficiency, scalability, security, real-time reactions, and smartness. This results in the requirement for exceedingly sophisticated system design and construction. Due to its resilient, scalable, and self-organized characteristics, swarm intelligence is a profitable paradigm for dealing with complex, rapidly changing, and dynamic situations. In this survey, swarm intelligence-based techniques applied to IoT have been analyzed. To face vulnerabilities present in IoT environments, social creatures' behavior has been a matter of study for the development of optimization algorithms. We focused on the most popular swarm intelligence algorithms: the classical particle swarm optimization (PSO) and the artificial bee colony (ABC). We included in the present work a wide range of papers that proposed solutions to IoT challenges based on the principles of the aforementioned algorithms. Concerning the IoT challenges, we address three main problems that can undermine the performance of these technologies, with a focus on optimizing network traffic, energy, and data.

As future works, we propose to extend the paper considering works based on a broader set of swarm intelligence algorithms and their application to solve both discussed and new IoT challenges.

To provide only a few examples, the authors in [67] proposed a hybrid intelligent optimization algorithm (HIOA) to reduce total energy usage in an IoT network. Fuzzy logic, a genetic algorithm, and the chicken swarm optimization (CSO) method are all used by HIOA to build the best possible clusters. The unbalanced CH distribution is addressed by the CSO, which also proves to be a fair network load.

An energy-efficient cloud-based Internet of Everything (EECloudIoE) framework is suggested in [68] with the goal of extending the lifespan and decreasing traffic of IoT environment. In this study, the multiple IoT networks are clustered using the wind driven optimization technique to maximize energy usage. The next step is to select an optimal cluster head (CH) for all clusters employing the firefly algorithm to help reduce the amount of traffic that will be stored in the cloud.

The integration of swarm intelligence with IoT opens up opportunities for solving complex problems in a decentralized, scalable, and efficient manner. The future direction of swarm intelligence in IoT is promising, with a focus on improving the following:

- Scalability: IoT devices have limited computational and storage capabilities, which can make it challenging to implement swarm algorithms that are scalable for large networks of devices.
- Real-time communication: swarm algorithms require efficient communication between devices, which can be difficult to achieve in real time in large-scale IoT networks.
- Energy efficiency: IoT devices have limited energy resources, so the swarm algorithms must be energy-efficient in order to preserve battery life.
- Privacy: swarm algorithms often collect and exchange data among devices, which can raise privacy concerns.
- Robustness: swarm algorithms need to be robust against network failures, faulty devices, and malicious attacks, which can be difficult to achieve in a distributed IoT environment.
- Integration with other technologies: swarm algorithms must be integrated with other advanced technologies, such as artificial intelligence and edge computing, to fully leverage their potential in IoT applications.

Despite these challenges, the use of these algorithms has a lot of potential for future development, such as in the areas of smart cities, smart homes, and autonomous systems.

## 5. Conclusion

The world around us is becoming more intelligent and more responsive thanks to the Internet of Things (IoT), which combines the digital and physical worlds. IoT may be applied in countless fields and has an impact on how we live our daily lives. The widespread use of mobile devices and Wi-Fi has made this technology possible. The network of IoT devices offers many undeniable benefits, like direct communication between separate devices without human interference, easy access to wide-scale and real-time data, and remote and continuous device control. Even though they are potential advantages, these new possibilities also present some challenges. Many obstacles are being imposed across multiple dimensions by the rapid growth of IoT-connected devices, including the limited lifespan of battery-operated devices, uneven load distribution, high transmission latency, and data security, complexity, and volume.

SI is a collection of nature-inspired searching techniques. Several animals in nature, including birds, fishes, and bees, exhibit group behaviors. The collective talents of a group are far more lively than the individual skills of its members. A simulation technique to model biological group intelligence is the SI algorithm. Several large-scale, dynamic, and multi-objective issues have been successfully handled via SI. In general, all SI-based metaheuristics have demonstrated beneficial adaptive features for solving optimization issues affecting IoT.

This work reviewed swarm intelligence (SI) algorithms to tackle three main IoT challenges: (i) routing protocols and CH selection, (ii) power management, and (iii) data management. Forthcoming research proposes to summarize papers that address other IoT issues considering a wider set of SI algorithms beyond those already considered in this work.

## Figures and Tables

**Table 1:** Routing protocols and CH selection algorithms based on SI.

SI algorithm	Paper	CH selection	Routing protocol
PSO	Vijayalakshmi and Anandan [32]	X	X
	Wang et al. [33]	X	X
	Rao et al. [34]	X	
	Tam et al. [35]	X	
	Wang et al. [36]	X	X
	Zhou et al. [37]		X
	Hasan and Al-Turjman [38]		X
	Luo et al. [39]		X

ACO	Wang et al. [40]	X	X
	Gajjar et al. [41]	X	X
	Kumar et al. [42]	X	X
	Sun et al. [43]		X
	Sun et al. [44]		X
	Rosset et al. [45]		X
	Mahalaxmi and Rajakumari [46]		X
	Said [47]		X
	Thapar and Batra [48]		X
	Maheshwari et al. [49]	X	X

ABC	Kumar et al. [42]	X	X
	Shokouhifar and Jalali [50]	X	X
	Reddy and Babu [51]	X	

**Table 2:** Power management algorithms based on SI.

SI algorithm	Paper
PSO	Pau et al. [52]
	Majdi et al. [53]
	Priyadarshi et al. [54]
	Hurtado et al. [55]

ACO	Lee et al. [56]
	Sharma and Grover [57]

ABC	Muhammad et al. [58]
	Yousefi et al. [59]
	Vijayashree and Dhas [60]
	Mann and Singh [61]

**Table 3:** Data optimization algorithms based on SI.

SI algorithm	Paper
PSO	Tsai et al. [15]
	Zhou et al. [62]
	Sung and Chiang [63]

ACO	Singh et al. [64]
	Chamoso et al. [13]

ABC	Ahmad et al. [19]

## Data Availability

No data were used to support this study.
